# Predictive value of FibroScan in detecting liver fibrosis in HBeAg negative patients with chronic hepatitis B whose HBV DNA 2000–20000 IU/ml with ALT 1–2 times the upper limit of normal and those with HBV DNA >20000 IU/ml and normal ALT

**DOI:** 10.14744/nci.2021.35545

**Published:** 2021-12-31

**Authors:** Nilay Danis, Ulus Salih Akarca, Ilker Turan, Zeki Karasu, Galip Ersoz, Funda Yilmaz, Deniz Nart, Aysin Zeytinoglu, Memnune Selda Erensoy, Fulya Gunsar

**Affiliations:** 1. Division of Gastroenterology, Department of Internal Medicine, Karabuk University Faculty of Medicine, Karabuk, Turkey; 2. Division of Gastroenterology, Department of Internal Medicine, Ege University Faculty of Medicine, Izmir, Turkey; 3. Department of Pathology, Ege University Faculty of Medicine, Izmir, Turkey; 4. Department of Medical Microbiology, Ege University Faculty of Medicine, Izmir, Turkey

**Keywords:** Fibrosis, HBV, liver biopsy, transient elastography

## Abstract

**Objective:**

In hepatitis B infection, it is difficult to make a treatment decision in patients with slightly elevated transaminases and HBV DNA level between 2000 and 20000 IU/ml, and in those with normal ALT, despite high levels of HBV DNA. Objectives: In HBeAg negative patients whose HBV DNA levels were between 2000 and 20000 IU/ml with ALT 1–2 times the upper limit of normal (ULN) and those with HBV DNA >20000 IU/ml and normal ALT, the concordance between liver fibrosis in biopsy and liver stiffness measured by transient elastography with FibroScan^®^ (FS) was investigated, and diagnostic value of FS to predict the liver fibrosis was tested.

**Methods:**

The patients were selected from the outpatient hepatology clinics between the dates of November 2014 and October 2016 among those who were taken liver biopsy. Transient elastography was obtained within 3 months after liver biopsy. The diagnostic value of FS in detecting advanced fibrosis or moderate to advanced (MTA) fibrosis was investigated for each group.

**Results:**

In 38 patients with HBV DNA 2000–20000 IU/ml and ALT 1–2×ULN, advanced fibrosis was detected in only one patient (2.6%) on liver biopsy, sensitivity of FS to show advanced fibrosis is 100%, specificity 78.3%, and diagnostic accuracy rate 79%. The area under curve was determined to be 0.892. In detecting MTA fibrosis, these values are 100%, 62%, 71%, and 0.810, respectively. Of 79 patients with HBV DNA >20000 IU/ml and normal ALT, five had advanced (5.5%) and 18 had MTA (23%) fibrosis. Sensitivity of FS in detecting advanced fibrosis was 100%, specificity 87.8%, and accuracy 88.6%, and these values for MTA fibrosis were 85.7%, 81%, and 82.3%, respectively.

**Conclusion:**

Because of false negativity in a few patients with HBV DNA >20000 IU/ml in detecting MTA, FS may be combined with other non-invasive techniques. Negative predictive values of FS in predicting advanced or MTA fibrosis were very high, while positive predictive values were low. However, FS may save several patients from liver biopsy.

**H**epatitis B infection is the leading cause of liver cirrhosis and hepatocellular carcinoma and the most common indication for liver transplantation in Turkey. The prevalence of HBsAg positivity across the country is around 4%. Although the incidence of HBV is gradually decreasing, new cases are still being detected and a significant proportion of patients develops liver complications.

With existing drugs, the hepatitis B virus can be effectively suppressed, liver complications can be avoided, and the liver histology improves. However, since the drugs cannot eradicate the virus and only suppress its replication, there is no real cure for the disease. The aim of treatment is, therefore, to suppress the virus and correct the liver disease. In this case, people whose HBV replication is naturally suppressed and whose liver is in good condition will not receive treatment. The patients to be treated should be selected on the basis of these facts. The two most important parameters in treatment decisions are the degree of HBV replication (HBV DNA level) and the stage of liver disease.

There is a consensus that treatment should be given regardless of liver impairment in patients with HBV DNA level >20000 IU/ml and ALT levels permanently higher than 2 times the upper limit of normal (ULN) [1, 2]. There is no predefined indication for the treatment of patients with HBV DNA levels between 2000 and 20000 IU/ml with normal or minimally elevated transaminases, or those with HBV DNA >20000 IU/ml and normal transaminase levels. The severity of liver diseases in these patients is crucial in the treatment decision. The most realistic way to understand the extent of liver damage is a liver biopsy. However, most of these patients have normal or minimal hepatitis in their liver biopsy, and treatment is not indicated yet. Therefore, to increase the diagnostic performance of liver biopsy, the patients to be biopsied can be selected by non-invasive techniques. Consequently, a significant proportion of patients may be spared from a morbid and rarely mortal invasive diagnostic method. There are several non-invasive techniques to estimate the level of liver fibrosis [3–5]. Most of them are based on blood tests such as simple APRI and Fib4 scores, as well as more complex test combinations. Another group of tests is derived from imaging techniques. One of the most common and easy to apply technique is the transient elastography performed with Fibroscan® (FS) [6, 7].

**Highlight key points**

• In this study, FS has been shown to save liver biopsy in a substantial proportion of Hepatitis B infected patients with slightly elevated transaminases and HBV DNA level between 2000 and 20000 IU/ml, and those with HBV DNA >20,000 IU/ml and normal ALT.
• However, a few false-negative results among patients with HBV DNA >20,000 IU/ml and normal ALT suggest that FS may be combined with other non-invasive techniques or may be repeated after some time.

In this study, we aimed to test the concordance between hepatic stiffness measured with FS and fibrosis on liver biopsy in HBeAg-negative patients with an HBV DNA level between 2000 and 20,000 IU/ml with an ALT level between 1 and 2 times the ULN and in those with HBV DNA >20,000 IU/ml whose ALT was normal. Based on this, the diagnostic value of FS was sought.

## MATERIALS AND METHODS

Patient selection: Among the newly diagnosed HBeAg-negative HBV carriers who were admitted to the hepatology outpatient clinic between November 2014 and October 2016 and who had a liver biopsy performed, the following patients were invited to the study:

1. Those with an HBV DNA level between 2000 and 20000 IU/ml and an ALT level between the ULN and 2 times the ULN. ALT elevation should have been proven 2 times, at least 3 months apart.
2. Patients with HBV DNA level >20000 IU/ml and ALT level not exceeding the ULN. It was required that ALT was shown to be normal 2 times at least 3 months apart.

Patients who had been treated for hepatitis B, those with concurrent liver disease including fatty liver, HIV infection, alcohol abuse, clinically significant cirrhosis, pregnant women, and heart failure were excluded from the study. Patients with insufficient liver biopsy were not included in the evaluation. This study was approved by Ege University Faculty of Medicine Ethics Committee with an approval number 20-7T/87. An informed consent was required.

FS measurements were taken no longer than 3 months after the date of the liver biopsy. FS measurements were made in accordance with known universal criteria.

### Definitions

Liver biopsies were evaluated by an experienced hepatopathologist and evaluated according to Ishak score. Accordingly, those with fibrosis 0–2 were classified as mild, those with 3–4 moderate, and those with 5–6 as advanced fibrosis. Based on our previous data, liver stiffness was categorized as <7.5 kPa mild, 7.5–10.1 kPa moderate, and >10.1 advanced fibrosis in FS measurement.

### Statistical Analysis

The compatibility of the stages in the liver biopsy with the FS stages was checked by Chi-square and Fisher’s exact test when appropriate. Accordingly, sensitivity and specificity of FS in indicating mild fibrosis, moderate to advanced (MTA) fibrosis, and advanced fibrosis were calculated. P<0.05 was considered significant in statistical tests. Evaluations were made separately for the patients whose HBV DNA 2000–20000 IU/ml and HBV DNA >20000 IU/ml. Area under curves (AUC’s) of the groups were compared using the DeLong’s technique [8]. MedCalc Statistical Software version 19.5.5 (MedCalc Software bvba, Ostend, Belgium) was used for statistical analysis.

## Results

Of 123 patients who met the criteria participated, 52 (42.3%) were female and 71 (57.7%) were male; mean age was 45.3±13.2 years. The mean HBV DNA levels were 5.29±2.08 log IU/ml, grade in liver biopsies was 5.74±2.30, and stage was 1.64±1.57. The liver biopsy grades and stages are shown in Table 1.

**Table 1.** Histopathologic findings in patients according to Ishak scoring system

Stage	Percent
Mild (n=89)	
0	31.7
1	21.1
2	19.5
Moderate (n=28)	
3	13.8
4	8.9
Advanced (n=6)	
5	2.4
6	2.4
Total (n=123)	100.0

Thirty-eight patients with HBV DNA 2000–20000 IU/ml and ALT between 1× and 2×ULN, 79 patients with HBV DNA >20000 IU/ml and normal ALT participated in the study. The distribution of the patients with advanced fibrosis in the liver biopsy and in the FS measurement among the patients with HBV DNA 2000–20000 IU/ml is shown in Table 2A. In this group of patients, only one patient (2.6%) had advanced liver fibrosis on the liver biopsy. Therefore, a 100% negative predictive value (NPV) of FS in detecting advanced fibrosis is already an expected finding. However, FS indicated advanced fibrosis in nine patients, which means an 11% positive predictive value (PPV). The diagnostic values of FS in detecting advanced fibrosis in the lower HBV DNA group are shown in Table 3A. Although its sensitivity and specificity and AUC value appear to be high, its PPV is very low.

**Table 2.** Distribution of liver fibrosis in liver biopsy and predicted fibrosis in FibroScan

A			
HBV DNA 2000–20000 IU/ml	Stiffness by FibroScan		Total
	Mild to moderate	Advanced
Liver biopsy			
Mild to moderate fibrosis	29	8	37
Advanced fibrosis	0	1	1
Total	29	9	38
B			
HBV DNA 2000–20000 IU/ml				Mild	Moderate to advanced	Total
Liver biopsy
Mild fibrosis	18	11	29
Moderate to advanced fibrosis	0	9	9
Total	18	20	38
C			
HBV DNA >20000 IU/ml	Mild to moderate	Advanced	Total
Liver biopsy			
Mild to moderate fibrosis	65	9	74
Advanced fibrosis	0	5	5
Total	65	14	79
D			
HBV DNA >20000 IU/ml				Mild	Moderate to advanced	Total
Liver biopsy			
Mild fibrosis	47	11	58
Moderate to advanced fibrosis	3	18	21
Total	50	29	79

**Table 3.** Diagnostic values of FibroScan in predicting liver fibrosis

	A		B		C		D	
	In patients with HBV DNA 2000–20000 IU/ml and ALT 1–2×ULN for advanced fibrosis	In patients with HBV DNA 2000–20000 IU/ml and ALT 1–2× ULN for moderate to advanced fibrosis	In patients with HBV DNA >20000 IU/ml and normal ALT for advanced fibrosis	In patients with HBV DNA >20000 IU/ml and normal ALT for moderate to advanced fibrosis
Sensitivity (%)	100.0	2.5–100.0	100.0	66.37–100.0	100.0	47.81–100.0	85.71	63.65–96.95
Specificity (%)	78.37	61.78–90.17	62.07	42.26–79.313	87.83	78.16–94.28	81.03	68.59–90.13
Area under curve (%)	0.89	0.74–0.96	0.81	0.65–0.91	0.94	0.86–0.98	0.83	0.73–0.90
Positive likelihood ratio (%)	4.62	2.50–8.54	2.63	1.65–4.2	8.22	4.45–15.16	4.51	2.58–7.91
Negative likelihood ratio (%)	0.0		0.0		0.0		0.17	0.06–0.50
Disease prevalence (%)	2.63	0.07–13.81	23.68	11.44–40.24	6.32	2.08–14.15	26.58	17.26–37.72
Positive predictive value (%)	11.11	6.34–18.75	45.0	33.93–56.58	35.71	23.14–50.61	62.06	48.31–74.12
Negative predictive value (%)	100.0		100.0		100.0		94.0	84.50–97.82
Accuracy (%)	78.94	62.68–90.44	71.05	54.09–84.57	88.60	79.47–94.65	82.27	72.05–89.95

HBV: Hepatitis B whose; ALT: Alanine aminotransferase; ULN: Upper limit of normal.

According to international guidelines, patients with HBV DNA level between 2000 and 20000 IU/ml are required to have MTA fibrosis in liver biopsy for commencing treatment. In the group of the patients with HBV DNA level 2000–20000 IU/ml and ALT 1–2×ULN, MTA fibrosis (F3–4: 8 and F5–6: 1) was detected in nine patients (24%) on the liver biopsy (Table 2B). Liver stiffness was found to be ≥7.5 kPa in all of these patients. Accordingly, none of the patients with mild fibrosis on biopsy had liver stiffness ≥7.5 kPa. However, MTA fibrosis was suggested in 20 patients with FS. If a biopsy decision was based on the findings of FS, 18 patients (47%) will be saved from liver biopsy, but biopsy would be performed in 11 patients even though there was no indication for treatment. According to the FS measurement, none of the patients who needed treatment were deprived of treatment. The diagnostic values of FS in detecting MTA fibrosis are shown in Table 3B.

Advanced fibrosis in the liver biopsy was found in 5 (6%) of 79 patients with HBV DNA >20,000 IU/ml and normal ALT (Table 2C). None of these patients had mild or moderate fibrosis with FS. FS has a superior diagnostic capability in ruling out advanced fibrosis in this group of patients. However, only 5 (36%) of 14 patients suspected advanced fibrosis with FS have advanced fibrosis in liver biopsy. Diagnostic accuracy for advanced fibrosis of FS in these patients was 88.6% with AUC: 0.939 (Table 3C). Sensitivity and specificity were high, but PPV still was low.

In patients with HBV DNA >20000 IU/ml and normal ALT, the most important parameters in the treatment decision are the level of hepatic fibrosis and the family history of HCC. Patients with MTA liver fibrosis should start treatment. The rate of MTA fibrosis among this group of patients was 21/79 (26.6%) (Table 2D). FS indicated the same degree of fibrosis in 18 (86%) of these patients. However, there was a discrepancy between FS and liver biopsy in three patients (14%), in whom liver biopsy showed MTA fibrosis, while FS indicated mild fibrosis. Of 58 patients with mild fibrosis in liver biopsy, 11 were reported as more advanced fibrosis in FS. There was a concordance between FS and liver biopsy regarding mild fibrosis in 47 of 50 (94%) patients. In other words, although FS has shown mild fibrosis, 6% of patients could have MTA fibrosis. In this case, these patients may be deprived of treatment if we adhere to the FS for biopsy indication. The diagnostic values of FS showing MTA fibrosis in patients with HBV DNA >20000 IU/ml and normal ALT are presented in Table 3D.

There was no statistically significant difference in indicating advanced fibrosis and MTA fibrosis of FS in patients with HBV DNA 2000–20000 IU/ml, although diagnostic accuracy and specificity appeared to be higher (Table 3). Likewise, in the group of patients with HBV DNA >20000 IU/ml, no statistical difference was found regarding the diagnostic value of FS between advanced and MTA fibrosis. Diagnostic values of FS in the patients with HBV DNA levels 2000–20000 IU/ml and HBV DNA >20000 IU/ml were not different, as well. PPVs appear to be different in distinct groups, yet they were not different statistically.

## Discussion

Nucleos (t) id analogs cannot eradicate HBV from the body and only suppress its replication. However, it improves liver histology and prevents liver complications. Therefore, there is no indication for treatment in cases where HBV is suppressed naturally and when the liver is healthy. Although the indication for treatment is very clear in some cases, the treatment decision is personalized mostly according to the liver histology in cases where the replication shows a borderline elevation and the liver damage is not evident despite significant replication. However, since liver biopsy is an invasive diagnostic method, it is not practical to use it commonly and especially for follow-up. For this reason, many non-invasive techniques are used to estimate the liver fibrosis. The most widely used one is FS, which works with the principle of transient elastography.

Application of FS in hepatitis B has been the subject of many studies, and a close correlation has been found between biopsy-detected fibrosis and FS measurements [9–13]. FS may discriminate HBeAg negative hepatitis B from HBeAg negative HBV infection [12, 14]. Higher stiffness was found to predict the development of liver complications [15, 16]. Liver stiffness has been demonstrated to decrease during the treatment of hepatitis B [17]. However, we target a specific population which the histology is highly decisive in treatment, namely HBeAg negative patients whose HBV DNA level from 2000 to 20000 IU/ml with ALT 1–2×ULN, and those with HBV DNA >20000 IU/ml and normal ALT. It is of great importance to detect advanced fibrosis for a close follow-up for liver complications, and to find MTA fibrosis to start the treatment. In this respect, FS seems not to be competent to detect the advanced fibrosis in patients with the lower HBV DNA levels, which had 11% PPV. In fact, this is not an unexpected result, since a very low rate of advanced fibrosis in this kind of patients (1/38) is inherent which leads to a 100% NPV and very low PPV. Because of this, despite a 100% NPV, a very much value should not be ascribed to FS. In patients with HBV DNA ≤20000 IU/ml and slightly elevated ALT, if MTA fibrosis is detected in the liver, treatment is appropriate. Therefore, the value of FS is important in terms of showing MTA fibrosis. In our patients, MTA fibrosis was found in liver biopsy in nine patients (24%). FS did not show mild fibrosis in any of these nine patients. If only the FS result is acted on, no patient who needed treatment was deprived of treatment with the FS result. However, FS gave false positive results for MTA fibrosis in 11 patients and liver biopsy was performed to these patients. However, according to all guidelines, it is strongly recommended to investigate the stages of liver disease in patients who already have HBV DNA 2000–20000 IU/ml if there are elevated transaminases. Since the gold standard for this purpose is liver biopsy, it can be said that FS saves liver biopsy in half of these patients. It seems that, it can be safely spared the patients from liver biopsy without the risk of treatment deprivation. However, to increase the detection rate of MTA, FS may be combined with other non-invasive techniques.

The other patient group for whom the evaluation of liver fibrosis is very important in the treatment decision is those with HBV DNA >20,000 IU/ml and normal ALT.

Another group of patients whose liver histology is decisive to treatment is that the patients with HBV DNA >20,000 IU/ml and normal ALT. In young HBeAg positive patients, this indicates HBeAg positive chronic infection, formerly immune tolerant phase of infection. Liver histology is almost always normal or near-normal; however, the age gets older the histology may be deteriorated, especially in HBeAg negative state. In our patients, 13 of 79 patients (16.5%) had moderate and 5 (6%) had advanced fibrosis. None of the patients with advanced fibrosis in biopsy had advanced stiffness, while false positivity was observed in nine patients. More importantly, to start treatment, MTA fibrosis should be differentiated from mild fibrosis. Sensitivity of FS was 85.7% and specificity was 81.0% in this respect. AUC was found to be 0.834 as an indicator of a considerable relationship. Of 21 patients with MTA fibrosis, 3 (14%) were labeled to have a mild fibrosis with FS which may lead to miss a few patients deserved to be treated. Because of this, FS should be combined with other non-invasive techniques before definite decision [6, 18]. To repeat, the elastography after a couple of month may be another alternative. On the other hand, FS yielded to save liver biopsy in 60% of the patients.

Several studies have reported a good correlation between liver stiffness and fibrosis in liver biopsy in hepatitis B. A meta-analysis demonstrated that as the degree of fibrosis increases, the sensitivity and specificity of the FS measurement in detecting the relevant degree of fibrosis increase [19]. A considerable difference in stiffness of the liver was reported in HBeAg negative HBV infection and HBeAg negative chronic hepatitis [12, 14]. Huang et al. [20] conducted a study examining chronic hepatitis B with ALT <2×ULN and investigating the diagnostic value of FS. However, patients with low-level (≤20,000 IU/ml) HBV DNA, which complicates the treatment decision, were not specifically selected in this study. Chen et al. [21] showed that FS predicted advanced fibrosis in patients with minimally elevated ALT, like our patients, but in HBeAg positive cases.

The limitation of our study is the small number of the studied population. A more accurate result of FS in predicting advanced fibrosis or MTA in gray zone patients would be revealed in studies with the higher patient numbers. Another point that is the common problem in FS studies is that the cut-off values are arbitrary. We defined cutoff values in our previous higher number of HBV infected patients, but a small change in these values can result in large changes in the predictive values of the measurements. Then, we cannot say that the selected cut-offs are the best values for this population. FS measurements are affected by inflammation of the liver, hepatic congestion, fatty infiltration, etc. Levels of transaminases and necro-inflammatory activity in the liver can affect liver stiffness [11, 22]. However, it was reported that in patients with ALT <2×ULN, ALT had no impact on hepatic stiffness [23]. In our study, patients were homogeneous with respect to transaminase levels and such interference is not expected.

### Conclusion

Patients with HBV infection whose decision to initiate treatment depends on liver histology, that is, patients with HBV DNA 2000–20000 IU/ml and ALT 1–2×ULN, and those with HBV DNA >20,000 IU/ml with normal ALT, FS has been shown to save liver biopsy in a substantial proportion of patients. No patient required treatment but was not treated due to FS measurement in patients with the lower HBV DNA. However, a few false negative results among patients with HBV DNA >20,000 IU/ml and normal ALT suggest that FS may be combined with other non-invasive techniques or may be repeated after some time.
